# Gaucher Disease and Gaucher Cells

**DOI:** 10.4274/tjh.2015.0043

**Published:** 2015-05-08

**Authors:** Sevgi Gözdaşoğlu

**Affiliations:** 1 Retired Professor of Pediatric Hematology and Oncology, Ankara, Turkey

**Keywords:** Gaucher cells, Electron microscopy

## TO THE EDITOR

I read the paper entitled “Gaucher cells or pseudo-Gaucher cells: that’s the question” written by Gören Şahin et al. in a recent issue of this journal. The authors mentioned the main findings of Gaucher cells and pseudo-Gaucher cells in their article without calling attention to the lysosomal enzyme β-glucocerebrosidase levels [1].

Gaucher disease is inherited as an autosomal recessive disorder resulting from mutations at the glucocerebrosidase locus on chromosome 1q21. In this disorder, glucosylceramide (glucocerebroside) is stored in the reticuloendothelial system due to a deficiency of the lysosomal enzyme β-glucocerebrosidase [2]. The storage and deposition of glucocerebroside within these cells, prominently macrophages, results in the appearance of Gaucher cells, which are very large cells with a diameter of 20-80 µm, round or polyhedral. Gaucher cells have small, usually eccentrically placed nuclei and cytoplasm with characteristic wrinkles or striations. Electron microscopy reveals that the cytoplasm contains spindle or rod-shaped membrane-bound inclusion bodies of 0.6-4 µm in diameter consisting of numerous small tubules of 13-75 nm in diameter. Electron microscopy allows the identification of all stages of formation of the inclusions [3,4].

**Figure 1:**
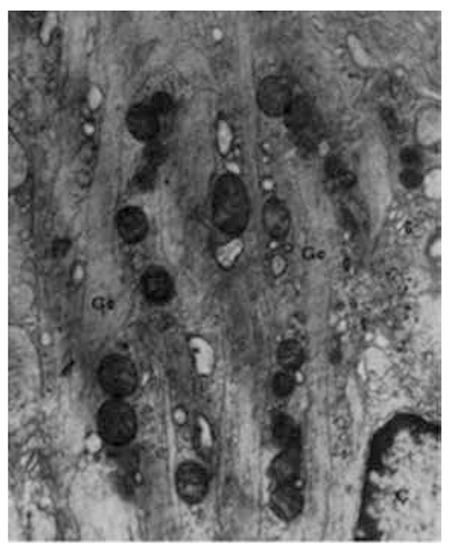
Electron micrograph of a Gaucher cell in the liver. In the cytoplasm, large Gaucher bodies containing tubular elements (25,000^x^, Gc: Gaucher bodies, Ç: nucleus, G: Golgi apparatus).

**Figure 2:**
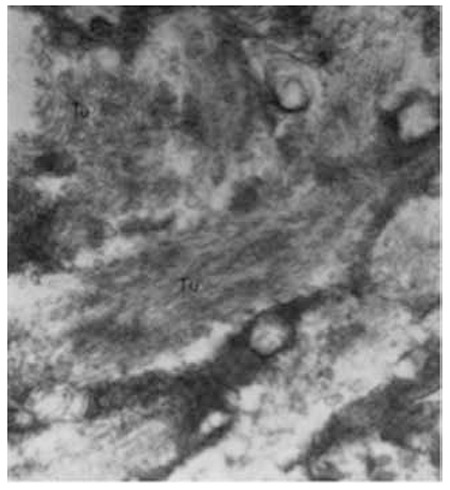
The cytoplasm of a Gaucher cell showed cytoplasmic bodies containing elongated tubular structures (72,000[ref:x]x[/ref], Tu: tubules).

Five patients were diagnosed with Gaucher disease by the presence of many Gaucher cells in the bone marrow associated with organomegaly and cytopenias between 1964 and 1970 in our department at Ankara University. Liver biopsy was performed in one of these patients and electron microscopic study was done. Ultrastructural analysis revealed many Gaucher bodies filled with tubules in the cytoplasms of Gaucher cells. These cells appeared as modified Kupffer cells by the accumulation in the cytoplasm of the cerebroside tubular material (Figures 1 and 2) [5,6].

While Gaucher cells are a hallmark of the disease, the appearance of these cells in the bone marrow is not pathognomonic because pseudo-Gaucher cells have been described in several other hematologic disorders including chronic granulocytic leukemia, Hodgkin’s disease, multiple myeloma, and AIDS [4,7]. Zidar et al. reported pseudo-Gaucher cells in the bone marrow of a patient with Hodgkin’s disease. The patient’s peripheral blood leukocyte β-glucosidase and serum acid phosphatase levels were elevated, and after 6 cycles of systemic chemotherapy, all signs of Hodgkin’s disease and pseudo-Gaucher cells disappeared [8]. The presence of pseudo-Gaucher cells can pose a diagnostic challenge. In this respect, it is of crucial importance to demonstrate enzymatic deficiency for the diagnosis of Gaucher disease [7].

**Conflict of Interest Statement**

The author of this paper has no conflict of interest, including specific financial interests, relationships, and/or affiliations relevant to the subject matter or materials included in this manuscript.
