# Supplementary material for: Gaucher Disease and Gaucher Cells
Source: Turk J Haematol. 2015 May 8;32(2):187–8. doi: 10.4274/tjh.2015.0043 (PMC4451492; doi:10.4274/tjh.2015.0043)
Supplement: Related texts

# Reply: Authors' Response

**Authors:** Olga Meltem Akay, Türkiz Gürsel

## REPLY

We read the recent letter by S. Gözdaşoğlu regarding our manuscript with great interest. We would like to thank the author for bringing lysosomal enzyme β -glucocerebrosidase levels into attention. We absolutely agree that Gaucher cells in the bone marrow is not pathognomonic for Gaucher disesase, since several hematological malignancies can present with pseudo-Gaucher cells in the bone marrow, like in our case report. Therefore, studying leukocyte β-glucosidase levels is very important in differential diagnosis. In our case, we did not study this enzyme level because the patient age, the presence of M band in serum electrophoresis and the presence of lytic bone lesions in the skull made the multiple myeloma diagnosis straightforward. However, we went through the published literature once again in this regard and realized that there were only about 50 articles published about pseudo-Gaucher cells. In few of them leukocyte β-glucosidase enzyme levels were reported [1,2]. When we reviewed these papers, we saw that these enzyme levels can change according to homozygosity or heterozygosity of the disease. Heterozygotes showed intermediate levels of enzyme activity. In a study by Pentchev et al. [1] enzyme activity was about 15% of normal in the adult nonneurologic form (type 1) and about 2.3% in the neurologic forms (types 2 and 3). The authors concluded that all 3 forms of Gaucher disease result from a structurally mutated enzyme with altered catalytic efficiency. Taken together, leukocyte b-glucosidase enzyme level is critical in the differential diagnosis of pseudo-Gaucher cell dilemma cases.

## References

[1] Pentchev PG, Neumeyer B, Svennerholm L, Groth CG, Brady RO (2183). Immunological and catalytic quantitation of splenic glucocerebrosidase from the three clinical forms of Gaucher disease. Am J Hum Genet.

[2] Zidar BL, Hartsock RJ, Lee RE, Glew RH, La Marko KL, Pugh RP, Raju RN, Shackneys E (1987). Pseudo-Gaucher cells in the bone marrow of a patient with Hodgkin’s disease. Am J Clin Pathol.
